# Genomic and genetic advances of oiltea-camellia (*Camellia oleifera*)

**DOI:** 10.3389/fpls.2023.1101766

**Published:** 2023-04-03

**Authors:** Changrong Ye, Zhilong He, Jiayu Peng, Rui Wang, Xiangnan Wang, Mengjiao Fu, Ying Zhang, Ai Wang, Zhixian Liu, Gaofeng Jia, Yongzhong Chen, Bingchuan Tian

**Affiliations:** ^1^ Academy of Innovation and Research, Huazhi Biotechnology Co. Ltd., Changsha, China; ^2^ Research Institute of Oil Tea Camellia, Hunan Academy of Forestry, Changsha, China; ^3^ Department of Research and Development, Mountain Yuelu Breeding Innovation Center, Changsha, China

**Keywords:** oiltea-camellia, genome, transcriptome, multi-omics, molecular breeding

## Abstract

Oiltea-camellia (*C. oleifera*) is a widely cultivated woody oil crop in Southern China and Southeast Asia. The genome of oiltea-camellia was very complex and not well explored. Recently, genomes of three oiltea-camellia species were sequenced and assembled, multi-omic studies of oiltea-camellia were carried out and provided a better understanding of this important woody oil crop. In this review, we summarized the recent assembly of the reference genomes of oiltea-camellia, genes related to economic traits (flowering, photosynthesis, yield and oil component), disease resistance (anthracnose) and environmental stress tolerances (drought, cold, heat and nutrient deficiency). We also discussed future directions of integrating multiple omics for evaluating genetic resources and mining key genes of important traits, and the application of new molecular breeding and gene editing technologies to accelerate the breeding process of oiltea-camellia.

## 1 Introduction

Oiltea-camellia (*Camellia oleifera* Abel.) belongs to Section *Oleifera* Chang Tax., Subgenus *Camellia* Chang Tax., *Camellia* L.*, Theaceae* Mirb. It is one of the most cultivated species in the *Camellia* genus. Its relatives include *Camellia sinensis* (the tea plant for drink) and *Camellia japonica* (ornamental flowers). There are five species in section *Oleifera*, including *C. oleifera*, *C. gauchowensis*, *C. lanceoleosa*, *C. sasanqua* and *C. vietnamensis*. In a broad sense, oiltea-camellia can be referred to the species with high oil content in *Camellia* genus, including *C. oleifera*, *C. meiocarpa*, *C.vietnamensis*, *C. yuhsienensis*, *C. reticulate*, *C. chekiangoleosa* and *C. semiserrata*. Among these species, *C. oleifera* is the most widely cultivated species for edible oil in China (Gao et al., 2011; Gong et al., 2020).

The species *C.oleifera* is also known as oil-Camellia, oiltea-camellia and oiltea tree. Oiltea-camellia is an important oil plant widely cultivated in many Asian countries, including the Philippines, Thailand, Japan and the Republic of Korea (Wang et al., 2013; Luan et al., 2020), and many provinces in Southern China, including Zhejiang, Hunan, Hubei, Jiangxi, Guangxi, and Guangdong (Lin et al., 2018). The seed kernel of *C. oleifera* contains up to 58% of high-quality edible oil, however, dry kernel oil content varies greatly among different cultivars from 23.1% to 57.7% (He et al., 2020a). The *C. oleifera* oil contained lots of compounds involved against bacteria, fungi and virus infection (Teixeira and Sousa, 2021). Oil extracted from the oiltea-camellia seeds is high in unsaturated fatty acids. The percentage of oleic acid in the oil was 76.0-81.4% (Yang et al., 2016), while the percentage in olive oil was 54.1-75.5% (Oğraş et al., 2016). Different oiltea-camellia cultivars have similar fatty acid compositions, including palmitic acid (C16:0, 7.68-10.01%), palmitoleic acid (C16:1, 0.14-0.55%), stearic acid (C18:0, 1.46-2.97%), oleic acid (C18:1, 75.78-81.39%), linoleic acid (C18:2, 4.85-10.79%), linolenic acid (C18:3, 0.30-1.11%), eicosenoic acid (C20:1, 0.68-0.97%), and tetracosenoic acid (C24:1, 0.08-0.36%)(You et al., 2019). Some of the health risks associated with consuming saturated fat can be avoided by replacing them with high oleic oil (Warner et al., 1997). Thus, oiltea-camellia is an important woody oil plant with high economic value. The planting area was approximately 4.3 million hectares in China in 2020 (Gong et al., 2022; Shen et al., 2022b). However, the breeding of oiltea-camellia is still very time-consuming because of its long life circle and complex genome. Recent advances in genomic and genetic studies of oiltea-camellia would speed up this process and improve the efficiency of breeding selection.

## 2 Genome of oiltea-camellia

Genomic assembly of oiltea-camellia is difficult because of its large and complex genome. The cultivated oiltea-camellia was inferred to be autotetraploid (2n =4x = 60) or autohexaploid (2n = 6x= 90) with high nuclear DNA content (2C-value=17.47 pg)(Huang et al., 2013; Qin et al., 2018; Ye et al., 2020). Fortunately, some wild diploid progenitors of cultivated oiltea-camellia with relatively small genome were identified. At the same time, with the decrease of sequencing cost and development of new bioinformatic analysis methods, some genomic and transcriptomic studies have been conducted, and chromosome-scale reference genomes of wild diploid oiltea-camellia species (*C. oleifera* var. “*Nanyongensis*”, *C. chekiangoleosa* and *C. lanceoleosa*) were assembled recently (Gong et al., 2022; Lin et al., 2022; Shen et al., 2022b). The genome sizes were similar but the numbers of genes identified in the three genomes were quite different. In the genome of *C. oleifera* var. *Nanyongensis*, totally 42,426 genes were annotated. In *C. chekiangoleosa*, 64,608 protein-coding genes were identified. And 54,172 genes were predicted in the *C. lanceoleosa* genome (**Table 1**). These high-quality reference genomes will greatly facilitate fundamental research on genomes of tetraploid and hexaploid varieties of oiltea-camellia.

**Table 1:** Comparison of assembled genomes of oiltea-camellia.

Assembly quality	*C. chekiangoleosa* (Shen et al., 2022b)	*C. oleifera* var. *Nanyongensis* (Lin et al., 2022)	*C. lanceoleosa* (Gong et al., 2022)
Genome size (Gb)	2.73	2.89	2.75
N50 of contigs (Mb)	1.92	1.00	1.20
N50 of scaffolds (Mb)	185.30	185.36	186.43
GC content (%)	39.23	37.51	40.55
Sequences anchored to chromosomes (%)	97.40	91.33	91.85
BUSCO (%)	93.60	90.10	95.42
LAI (%)	11.53	–	12.45
Heterozygosity rate (%)	–	2.52	2.20
Number of predicted genes	64608	42426	54172

BUSCO, benchmarking universal single-copy orthologs; LAI, long-terminal-repeat assembly index.

The species *C. chekiangoleosa* and *C. sinensis* had an ancient whole genome duplication (WGD) event, and a recent WGD event was shared among the species of genus *Camellia* (Shen et al., 2022b). Genes related to fatty acid synthesis were found to expand in both *C. chekiangoleosa* (Shen et al., 2022b) and *C. lanceoleosa* genome (Gong et al., 2022). This may account for the high oil content in both species. Genes related to linoleic acid synthesis were found to contract throughout evolution in the genome of *C. chekiangoleosa* (Shen et al., 2022b). The contracted genes in the genome of *C. lanceoleosa* were enriched in response to auxin and plant hormone signal transduction (Gong et al., 2022). Genes related to unsaturated fatty acid biosynthesis (*CchFAD2A*, *Cch15G000175*; *CchFAD2B*, *Cch10G003830*; *CchSAD2*, *Cch05G001837*), oil content (*SDP1*, *IAA26*, *FabD*, *Oleosin3*), fatty acid content (*SAC8*, *KASIII*, *SAD1/6*), oil biosynthesis (*ACC*, *SAD*, *DGAT*, *PDAT*, *G3PDH*) were identified (Gong et al., 2022; Lin et al., 2022; Shen et al., 2022b). However, the function and breeding application of these genes should be further validated. Three chromosome-level reference genomes of diploids are not enough to understand the overall genomic structure of oiltea-camellia. With the advances in bioinformatic analysis of big data, more reference genomes of the polyploids of oiltea-camellia are expected in the near future.

## 3 Genes for economic traits of oiltea-camellia

### 3.1 Flowering and pollination

Oiltea-camellia is a perennial plant with long juvenile phase. It takes more than three years before entering the reproductive phase. The mechanism of juvenile-to-adult transition was unknown. Two RAV homologs controlling seasonal flowering and juvenility were identified in loquat (Peng et al., 2021). The juvenile period of Arabidopsis was prolonged about three times when genes *EjRAV1* and *EjRAV2* were overexpressed (Peng et al., 2021). The decreased level of microRNA miR156/157 may induce the expression of floral integrators such as *FT*, *SOC1*, *AP1*, *LFY*, and *SPL* transcription factors to promote flowering (Wang, 2014; Xu et al., 2016; Jia et al., 2017; Xu et al., 2018). Future study on these genes in oiltea-camellia may help to understand the flower initiation and seasonal flowering in the early reproductive phase, and develop new strategy for breeding.

The floral induction of *C. oleifera* generally occurs from late April to early May, while the flowering occurs between later autumn and early winter (Wang et al., 2011). The lack of insects and other vectors during the flowering period leads to low fruit setting. The advance of the flowering time may increase fruit set and oil yield. The expression of *CoFT1*, a gene belonging to the flowering locus T family, changed with diurnal rhythms under different day-length conditions. Overexpression of *CoFT1* in Arabidopsis leaded to precocious flowering possible by increasing the expression of flowering related genes, such as *SOC1*, *AP1*, and *LFY* (Lei et al., 2017). *EMF2* was found to inhibit flowering during plant development (Chou et al., 2001; Zhou et al., 2021). Although the sequence of *CoEMF2* identified in *C. oleifera* was highly conservative compared with *EMF2* in other plants. The function of *CoEMF2* in *C. oleifera* was not explored (Jia et al., 2017; Peng et al., 2021).

Anthocyanins are the main pigment in flowers and fruits of plants (Wang, 2014; Xu et al., 2016; Jia et al., 2017; Xu et al., 2018), which can not only attract pollinators, but also filter ultraviolet rays to resist pathogens and improve plant fertility (Liu et al., 2018). Transcriptomic analysis of leaf buds, mature leaves, flower buds, flowers, immature fruits, and blackening seeds of *C. reticulata* identified that *MYBA1-a* and some anthocyanin biosynthesis related genes in the *FlaBS* pathway were highly expressed in flower buds and flowers (Yao et al., 2016). There are nine genes (*ANS*, *CHI*, *CHS1*, *CHS2*, *CHS3*, DFR*, F3H*, *PAL* and *UFGT*) involved in anthocyanin biosynthesis in *C. chekiangoleosa* (Wang et al., 2014). These genes may play important roles in anthocyanin biosynthesis during flower development, and increased anthocyanin may contribute to attract pollinators and improve pollination efficiency.

Furthermore, oiltea-camellia is a self-incompatible plant, which makes the variety development more complex. To achieve high yield of seeds, breeders have to develop two synthetic lines that can pollinate to each other and planting them together in the field. Genes related to self-incompatibilty have been identified in some plants such as grapevine, potato, pummelo, Arabidopsis, Brassica, Petunia, and *Camellia sinensis*, a relative species of *C. oleifera* (Tsuchimatsu et al., 2010; Kubo et al., 2015; Takada et al., 2017). Twelve homologous genes of ribonuclease T2 that similar to S-RNases gene of *C. sinensis* were identified in *C. lanceoleosa* (Gong et al., 2022) Self-pollination possibly induced the expression of serine carboxypeptidase-like (*SCPL*) and UDP-glycosyltransferase (*UGT*) and their encoded and interacting proteins, which increased the galloylated catechin level and lead to self-incompatibilty in *C. oleifera* (Chang et al., 2022). Recently, self-compatible plants of potato and self-incompatible *Brassica napus* have been created by gene editing (Ye et al., 2018). It is worth comparing the homologous genes of self-incompatability in oiltea-camellia, and validate some key genes by gene editing. The improvement in self-compatability will greatly benefit the variety development and cultivation of oiltea-camellia.

### 3.2 Photosynthetic efficiency

Photosynthesis, a process of light capture and carbon fixation, played key roles in crop yield (Long et al., 2006). Transcriptome analysis of the leaf of *C. oleifera* identified 12 genes (*rbcL, rbcS*, *PGK*, *PEPC*, *PLR*/*PYL*, *PP2C*, *SnRK2*, *PHYB*, *PIF3*, *GI*, *WRKY2*, *WRKY70* and *MYB44*) associated with photosynthetic efficiency by comparing gene expression in different groups with different photosynthetic efficiency. Three co-expression networks and ten connected genes that play crucial roles in the regulatory network of photosynthesis were also identified (He et al., 2021). The differences of photosynthesis among oiltea-camellia cultivars might be controlled by multiple genes. The photosynthesis efficiency of more genetic resources should be evaluated, and related genes should be validated for future breeding applications.

Rubisco is an key enzyme determining net photosynthesis by catalyzing CO_2_ fixation and ribulose diphosphate oxygenase reaction (Andersson and Backlund, 2008). The expression of two rubisco subunit genes, *CorbcL* and *CorbcS*, were strongly associated with oil yield. It was suggested that *CorbcL* and *CorbcS* can be used as candidate molecular biomarkers for selecting high oil yield cultivars (Chen et al., 2015).

### 3.3 Fruit abscission and fruit size

Fruit abscission occurred in the abscission zone during fruit ripening under environmental stresses (Osborne and Morgan, 1989; Bleecker and Patterson, 1997). The flower and fruit abscission rates of *C. oleifera* were high in many varieties, resulting in a decline in seed yield and becoming a major constraint for the commercial cultivation of *C. oleifera* (Chen et al., 2016). Ethylene is an important phytohormone regulating the fruit abscission of oiltea-camellia. In the abscission zone of abnormal fruits, the 1-aminocyclopropane-1-carboxylic acid (ACC) content increased significantly (Hu et al., 2021). The expressions of genes *CoACO1* and *CoACO2* increased significantly in the abscission zone of abnormal fruits (Hu et al., 2021). These genes were critical in ethylene regulation. Genes *CoIDA1*, *CoIDA2* and *CoIDA3* that control floral organ abscission in plants were also related to fruits abscission in *C. oleifera*. The expressions of genes *CoIDA1* and *CoIDA2* increased significantly in abscission zones of abnormal fruits of oiltea-camellia (Yang et al., 2021). It is possible to lockout these genes to decrease their expression and increase fruit setting rate.

Fruit size of oiltea-camellia directly related to the seed and oil yield. The expressions of 21 hub transcription factors were related to the fruit vertical diameter, horizontal diameter and volume of the fruit. Among these genes, the expressions of *SPL4*, *KLU*, *ABI4* and *YAB1* were significantly associated with these fruit traits (Ji et al., 2022). In addition, the fruit size of oiltea-camellia is also associated with the number of ploids in the genome, possibly due to the increased expression of the genes controlling fruit size. This phenomenon has been observed in many species, and successfully used in the breeding of some fruit plants like Kiwi (Wu et al., 2012). Most of the oiltea-camellia cultivars are autotetraploid or autohexaploid, which is a result of long-term selection for big fruit size and high yield. Polyploid induction and crossing between polyploids are still important strategy in oiltea-camellia breeding.

### 3.4 Oil yield and oil component

Oil yield is one of the most important traits for oiltea-camellia breeding. Oil yield can be estimated by dry kernel oil content and fresh fruit oil production rate. Dry kernel oil content ranges from 23.10% to 57.68%, while the fresh fruit oil production rate ranged from 1.49% to 12.91% among different cultivars (He et al., 2020b). The process of oil biosynthesis included various genes in different pathways. Gene *WRI1* and transcriptional factors *MYB* and *ZIP* were interacted with other genes and affected the oil synthesis (Gong et al., 2020). Understanding the molecular mechanism underlying oil biosynthesis, especially fatty acids biosynthesis, will facilitate the breeding of cultivars with high oil yield.

The glycerol-3-phosphate synthesis in *Saccharomyces cerevisiae* was promoted by high expression level of the glycerol-3-phosphate dehydrogenase 1 (*GPD1*) gene, resulting in an increase of oil content in the seeds (Remize et al., 2001). The *DGAT* gene has two non-homologous transcripts (*DGAT1* and *DGAT2*) that catalyzes the conversion of diacylglycerol (DAG) into triacylglycerol (TAG), thus, it is important for TAG biosynthesis (Lung and Weselake, 2006; Wang et al., 2006). The expression of DGAT unigenes were consistent with oil accumulation in developing seeds of oiltea-camellia (Lin et al., 2018). The expressions of *GPD1*, *DGAT1* and *DGAT2* were significantly higher in high oil-content seed compared with low oil-content seed (Wu et al., 2019). It was suggested that the coordinated high expression of genes *GPD1*, *DGAT1* and *DGAT2* promoted lipid biosynthesis and accumulation in the seed of high oil-content varieties.

Genes *EAR* (enoyl-ACP reductase), *HAD* (3-hydroxyacyl-ACP dehydrase), *KAR* (β-ketoacyl-ACP reductase) and *KASI* (β-ketoacyl-ACP synthase I) mainly regulate the biosynthesis of C16:0-ACP, which is a precursor of C18 fatty acids (Wang et al., 2003; Wang et al., 2011). Genes *FATA*, *FATB*, *KASII* and *SAD* were involved in the regulation of carbon chain length and saturation of fatty acid. The expression of *FATA* was consistent with the increase of oleic acid content during the seed development of oiltea-camellia (Lin et al., 2018). Gene *FATB* (palmitoyl-acyl-ACP thioesterase) mainly regulates the conversion of 16-carbon palmitoyl-ACP into palmitic acid (Dormann et al., 2000). The expressions of genes *EAR*, *HAD* and *KASI* were consistent with the level of C16:0-ACP during the seed development, while the tendency of the expression of *FATB* was contrary (Wu et al., 2019). Gene *KASII* (β-Ketoacyl-ACP-synthase II) encodes a key enzyme that catalyzes the conversion of C16:0-ACP into stearic acid, thus, the gene expression levels of *KASII* are closely associated with the stearic acid content in the seeds (Ye et al., 2009). The increased expression of the *KASII* gene during seed development promoted the biosynthesis of stearic acid, and provided resources for the biosynthesis of oleic acid (Wu et al., 2019).

In the fatty acid biosynthetic pathway, gene *SAD* (Stearoyl-ACP-desaturase) mainly catalyzes the desaturation of stearic acid (C18 fatty acid) to form oleic acid (C18:1)(Ye et al., 2009). The change in the expression of *SAD* gene was consistent with the accumulation of oleic acid (C18:1) (Wu et al., 2019). Gene *FAD2* (Fatty acid desaturase 2) mainly regulates the desaturation of oleic acid to form linoleic acid (Sivaraman et al., 2004). High expression level of *SAD* and low expression level of *FAD2* were critical to achieve high 18:1 fatty acid content in oiltea-camellia seeds, and the lipid biosynthesis pathway and regulatory mechanism of oil accumulation of *C. oleifera* was proposed (Lin et al., 2018). Genes *FAD3*, *FAD7* and *FAD8* are the key regulators for the conversion of linoleic acid into linolenic acid (Wu et al., 2019). The decreased expression of these genes in the later seed development stage also contributed to the accumulation of oleic acid (C18:1) (Wu et al., 2019). Two Indels and 362 SNPs in four key genes (*CoSAD1*, *CoSAD2*, *CoFAD2-A* and *CoFAD2-B*) related to unsaturated fatty acids biosynthesis were used to identify the association between genetic variants and oil content and quality including eight traits of fatty acid composition, a total of 90 associations were significant in the discovery group, and six of them were successfully validated in the validation group (Lin et al., 2019). The expression of genes *CoFBA* and *CoSAD2* were correlated with oil content, and the expression level of gene *CoFAD2* was correlated with fatty acid composition in the oiltea-camellia seeds (Zeng et al., 2014).

MicroRNAs (miRNAs) are important in mediating the post-transcriptional regulation of gene expression. Previous study showed that miRNAs were involved in lipid metabolism and seed development. By comparing the high and low oil content cultivars of oiltea-camellia, fifty-five deferentially expressed miRNAs were identified, among them, 34 miRNAs were up-regulated, and 21 miRNAs were down-regulated (Wu et al., 2021). In another study, twenty-three miRNAs regulating 131 target genes were identified, which was related to lipid metabolism process including the biosynthesis, accumulation and catabolism of fatty acid (Feng et al., 2017). Furthermore, the proteins involved in lipid metabolism and flavonoid biosynthesis were down-regulated in self-pollinated pistils (He et al., 2020b). However, the regulatory roles of these miRNAs were not well investigated.

## 4 Genes for abiotic stress tolerance and biotic stress resistance

### 4.1 Drought tolerance

Drought is considered as the most significant environmental factor in agriculture limiting the productive areas of the world (Kudo et al., 2017). Drought causes declining in crop yield and plant death in severe cases (Wang et al., 2003). The leaf osmotic adjustment substances, stomatal morphology and growth state were significantly affected by drought stress (Jaleel et al., 2009). Although oiltea-camellia is considered as drought-tolerant, its cultivation would be promoted by understanding the molecular mechanisms of drought tolerance and develop new drought-tolerant varieties, especially in the areas with serious water shortage. By transcriptomic sequencing of leaf samples of seedlings exposed to drought treatment, large number of genes were identified as deferentially expressed genes (Shen et al., 2022a). In a drought-tolerant cultivar, there were 124, 113, and 67 genes up-regulated after drought stress for 12, 24, and 36 hours, while in the drought-sensitive cultivar, there were152, 109, and 97 genes up-regulated after drought stress (Dong et al., 2017). Another transcriptomic study also showed that miR398 and miR408 were involved in the regulatory network of drought tolerance in oiltea-camellia (Huang et al., 2022). A gene encoding Reveille1 (*RVE1*) expressed differently between drought tolerant and susceptible varieties of *C. oleifera* after drought treatment (Huang et al., 2022). However, further study of these genes involved in metabolic pathways related to drought stress should be carried out to discover novel genes controlling drought tolerance of oiltea-camellia.

### 4.2 Extreme temperature tolerance

Temperature, especially low temperature, is one of the most important ecological factors affecting the productivity and survivability of oiltea-camellia plants (Theocharis et al., 2012). Screening cultivars for cold tolerance and high yield will help to increase the planting area and oil production. When comparing the gene expressions at 6 °C low temperature with a normal temperature of 25 °C, twelve genes (*CoLHCB5*, *CoARR-A*, *Coglgc*, *CoSNRK2*, *CobglB*, *CoFLS*, *CogalA*, *CoamyB*, *CoPAL*, *CopsbS*, *CoCYP73A* and *CoRafs2*) were identified to be differently expressed in mature leaves of *C. oleifera* by transcriptome sequencing and qRT-PCR (Theocharis et al., 2012). Another 12 deferentially expressed genes were also validated by qRT-PCR (Wu et al., 2020). When the environmental temperature decreased to 2 °C, the expression of C-repeat binding factor (*CBF*) gene was significantly increased in leaves (Chen et al., 2017). Genes related to cold acclimation and cold tolerance may be involved in transmembrane transporter activities.

The oiltea-camellia is relatively susceptible to high temperature, with an optimum mean temerature of 14-22 °C. The heat tolerance of *Camellia japonica* (flower) cultivars could be effectively evaluated under heat stress of 36-38 °C for one week (Li et al., 2006). There are significant differences in heat tolerance of *C. oleifera* cultivars evaluated by using semi-lethal temperature (LT50). The LT50 of 25 C*. oleifera* cultivars ranged from 45 to 57 °C (He et al., 2012; Wang et al., 2012). The leaf relative water content under heat and drought stresses was significantly correlated the expression levels of genes *Co-rbcL* and *Co-rbcS* (Wang et al., 2015). Over expression of these genes may help to improve the survivability and productivity of oiltea-camellia cultivars under climate change.

### 4.3 Disease and pest resistances

Plant diseases and pests are the predominant limiting factors for the industrial development of oiltea-camellia. The most serious camellia diseases and pests are camellia dieback and canker (caused by fungus *Glomerella cingulata*), flower blight (caused by fungus *Ciborinia camelliae*), leaf gall (caused by fungus *Exobasidium camelliae*), root rot (caused by fungus *Phytophthora cinnamomi*), alga leaf spot (caused by *Cephaleuros virescens*), tea scale (*Fiorinia theae*), cottony camellia scale (*Pulvinaria floccifera*) and camellia aphid (*Toxoptera aurantii*). There were seven main diseases identified on the trees of *C. oleifera* in Guangdong Province (Yan et al., 2021). Some disease-resistant species or cultivars have been selected in recent years. For example, species *C. yuhsienensis* is resistant to anthracnose, root rot diseases, and root knot nematode, but the high-yielding cultivar “Huashuo” of *C.oleifera* is susceptible to these diseases (Yang et al., 2004; Tan et al., 2011; Wei et al., 2013; Zhu et al., 2020; Chen et al., 2022b). Further study showed that the abundant and diverse microbial community in *C. yuhsienensis* rhizosphere may help to protect the host from pathogens (Li et al., 2021a)

The plant growth and seed yield of oiltea-camellia are affected by anthracnose, a disease caused by *Colletotrichum gloeosporioides*. Anthracnose resistance is one of the most important traits for variety development of oiltea-camellia. A cutinase gene *CglCUT1* encoded a cutinase has positive effect on fungal virulence of *C. gloeosporioides* on oiltea-camellia (Wang et al., 2017). Several studies focused on genes of *Colletotrichum fructicola* to identify potential fungicide targets for anthracnose control, and three genes (*CfSnf1*, *ScGcn5* and *CfVAM7*) were identified as critical factors for the fungi growth and pathogenicity (Zhang et al., 2019; Li et al., 2021b; Zhang et al., 2021). The metabolism pathway of purine in *C. fructicola* may contribute to its strong pathogenicity (Tan et al., 2021). By transcriptomic and metabolomic analyses of oiltea-camellia, key transcripts and metabolites associated with anthracnose resistance were identified, including 5001 deferentially expressed genes (DEGs) and 68 deferentially accumulated metabolites (DAMs). Further analysis of these DEGs and DAMs showed that arachidonic acid, epicatechin and procyanidin B2 are important for the anthracnose resistance of oiltea-camellia. A number of 479 deferentially expressed genes were significantly enriched in pathways of tyrosine metabolism and biosynthesis of flavonoid, isoquinoline alkaloid and phenylpropanoid (Yang et al., 2022). The biosynthesis of flavonoid might directly affect the anthracnose resistance of oiltea-camellia.

### 4.4 Nutrient deficiency

Soil nutrient is critical for plant growth and development. Nutrient deficiency causes stunted plant growth and low yield. However, very few studies on nutrient deficiency have been carried out for oiltea-camellia, especially on genetic perspectives. The oiltea-camellia plants have extraordinary Mn accumulation and toleration abilities, and proper application of nitrogen and potassium could enhance the efficiency of Mn phytoremediation (Li et al., 2019; Yu et al., 2019; Yu et al., 2020). Based on the analysis of degradome, transcriptome and small RNA data, thirty-two deferentially expressed miRNAs under low inorganic phosphate treatment, and three hub target genes (*ARF22*, *SCL6*, and *WRKY53*) controlling transcriptomic regulation of low inorganic phosphate stress tolerance were identified (Chen et al., 2022a). More studies on genetics of nitrogen, phosphate, potassium and other nutrients use efficiency are needed for understanding the molecular mechanism and for better breeding and production strategies.

## 5 Future perspectives

### 5.1 Studies on genetic resources and genome of oiltea-camellia

Oiltea-camellia is widely distributed in China and many Asian countries. For example, more than 1900 genetic resources of *C. oleifera* and its relatives from different countries and different provinces in China have been collected and conserved in the genebank at the Hunan Academy of Forestry. More than 400 accessions have been conserved in Hainan province. And more than 360 varieties have been registered in China. However, only very few species or accessions have been characterized and studied. It is urgent to carry out accurate phenotyping and genotyping studies of the conserved genetic resources, to build a database with multi-omics datasets, and to select a core collection for future gene mining and breeding applications. Genome-wide association study (GWAS) of genotyping and phenotyping data could also be used to identify genes controlling important traits. The screening of a large number of genetic resources to identify unique traits and alleles will enhance the availability of variation for breeding.

The genomes assembled are all diploid species of *Camellia*. Future sequencing and assembling the genomes of cultivars of autotetraploid and autohexaploid will greatly benefit the studies on genomic variations and gene identification. The large proportion of repetitive elements, high heterozygosity and the similarity of homologous chromosomes make it difficult to assemble the genomes of autopolyploids, however, with the development of new computing methods, genomes of some autopolyploid species such as potato have already been assembled (Bao et al., 2022; Sun et al., 2022; Wang et al., 2022). This will provide new solutions for future genomic studies on oiltea-camellia.

### 5.2 Studies on multi-omic solutions

As reviewed in this paper, there were some studies on the genome, transcriptome and metabolome of oiltea-camellia. However, these studies only focused on very few accessions or specific traits. The development of ‘omics’ technologies can generate different datasets for gene mining and breeding selection. High throughput phenotyping technologies (phenome) such as visual data collection using a drone will accelerate trait identification of the genetic resources. Comprehensive analysis of multi-omic data such as phenome, genome, transcriptome, metabolome, lipidome, proteome, and envirionome will enhance our understanding of the gene interaction and metabolic pathways of interesting traits. Multi-omic tools and approaches give more significant prospects to explore the function of important genes, to accelerate variety development, and to increase oil productivity in the future.

### 5.3 Application of molecular breeding technologies

Oiltea-camellia is a perennial tree crop with long juvenile phase, and the self-pollination is highly incompatible, which makes the breeding process more complex and time consuming. Although more than 2300 SSRs and 20200 SNPs were identified in oiltea-camellia (Xia et al., 2014), and a linkage map was made from 300 SNPs (Lin et al., 2022), current breeding for varieties with high yield and good quality remains challenging due to the lack of genomic and genetic information of the target genes and complex genetic background. Without high quality reference genome, application of molecular breeding tools is limited. Although previous studies identified various genes involved in different phenotypes of oiltea-camellia, most of them only explored the functions of these genes based on their expression in different tissues or under different stresses. The true function of these genes still need to be explored and validated. With the rapid advances in ‘omics’ technologies, big datasets will be generated and used for variety improvement. Integration of multi-omics data will accelerate the identification of genes and pathways responsible for important agronomic traits. High-throughput genotyping technologies such as genotyping by sequencing and high-density SNP chips could be used to screen large number of germplasm resources, and to identify novel allele variations. The understanding of the genes and pathways could provide opportunity to design idea varieties with superior agronomic traits through molecular breeding (Kumar et al., 2015). Furthermore, the whole-genome selection method is a promising approach for breeding selection of plants with complex genome and long life cycle like oiltea-camellia. Whole-genome selection is based on models from genotyping and phenotyping data of a reference population, which can increase the genetic gain of the target traits efficiently in the breeding populations. Marker-assisted selection and genomic selection would significantly increase the efficiency of breeding selection and shorten the breeding cycle, and should be practiced in the breeding process of oiltea-camellia.

## Author contributions

CY, ZH, JP and RW wrote the manuscript, XW, MF, YZ, AW and ZL provided references, GJ, YC and BT supervised the writing and reviewed the manuscript. All authors contributed to the article and approved the submitted version.
